# Longitudinal Changes in Neural Connectivity in Patients With Internet Gaming Disorder: A Resting-State EEG Coherence Study

**DOI:** 10.3389/fpsyt.2018.00252

**Published:** 2018-06-07

**Authors:** Sunyoung Park, Hyera Ryu, Ji-Yoon Lee, Aruem Choi, Dai-Jin Kim, Sung Nyun Kim, Jung-Seok Choi

**Affiliations:** ^1^Department of Psychiatry SMG-SMU Boramae Medical Center, Seoul, South Korea; ^2^Department of Psychiatry, Seoul St. Mary's Hospital, College of Medicine Catholic University of Korea, Seoul, South Korea; ^3^Department of Psychiatry Seoul Medical Center, Seoul, South Korea; ^4^Department of Psychiatry and Behavioral Science Seoul National University College of Medicine, Seoul, South Korea

**Keywords:** internet gaming disorder, EEG, coherence, fast frequency band, treatment response

## Abstract

**Aims:** The present study investigated neural connectivity associated with treatment responses in patients with Internet gaming disorder (IGD) using resting-state electroencephalography (EEG) coherence analyses.

**Methods:** We included 30 patients with IGD and 32 healthy control subjects (HCs). Of the IGD patients, 18 completed an outpatient treatment that included pharmacotherapy with selective serotonin reuptake inhibitors for 6 months. Resting-state EEG coherence and self-report questionnaires were used to evaluate clinical and psychological features pre- and post-treatment, and data were analyzed using generalized estimating equations.

**Results:** Compared with HCs, patients with IGD showed increased beta and gamma intrahemispheric coherence and increased delta intrahemispheric coherence of the right hemisphere at baseline. After 6 months of outpatient management, patients with IGD exhibited improvements in IGD symptoms compared with baseline, but they continued to show increased beta and gamma intrahemispheric coherence compared with that of HCs. No significant EEG coherence changes between the pre- and post-treatment assessments were detected in any band in the IGD group.

**Conclusion:** These findings suggest that significantly greater intrahemispheric fast-frequency coherence may be an important neurophysiological trait marker of patients with IGD.

## Introduction

Internet gaming disorder (IGD) is characterized by a pattern of excessive and repetitive use of Internet-based games (1). IGD has received increasing attention due to various negative consequences affecting normal daily life, academic and job performance, and psychological functioning (1, 2). Patients with a behavioral addiction, such as IGD, share certain clinical features, including impulsivity, craving, and the inability to control harmful behavior (3, 4). Recent studies have used neuroimaging and neurophysiological techniques to investigate the structural and functional changes in the brain associated with impulsivity or response inhibition to enhance our understanding of the characteristics of IGD (5–7).

Several neuroimaging studies have investigated the dysfunctional connectivity in patients with IGD. For example, Zhang (8) reported a decreased amplitude of low fluctuations in the orbitofrontal cortex and posterior cingulate cortex in young adults with IGD compared with controls. They also found that patients with IGD exhibited enhanced interactions in the default mode and executive control networks compared with controls. Additionally, patients with IGD showed increased connectivity in sensorimotor brain networks and altered interhemispheric resting-state functional connectivity in the prefrontal lobe, including the bilateral superior frontal gyrus, inferior frontal gyrus, and middle frontal gyrus (9, 10). These findings suggest that patients with IGD have impairments in reward-related processing, general cognitive functioning, and impulse control.

Although neuroimaging studies have identified the brain structures involved in resting-state activities, they provide limited information in terms of the temporal dynamics of neural networks in the brain. Electroencephalographic (EEG) coherence is useful for measuring abnormalities in functional brain organization with high temporal resolution (11). EEG coherence measures the consistency of phase differences in two brain regions and reflects the synchronization between neural populations and cortical connectivity (12). Increased coherence between two EEG electrodes suggests functional integration of two brain regions, whereas decreased coherence reflects the unrelated activities of two neural populations (13, 14).

A few studies that have investigated brain connectivity using resting-state EEG have reported that adolescents with an Internet addiction showed increased gamma coherence among the parietal, right temporal, and occipital areas compared with healthy controls (HCs) (15). Patients with IGD also exhibited enhanced intrahemispheric gamma coherence compared to controls (16). Furthermore, increased intrahemispheric connectivity within the fronto-temporal area may be associated with repetitive online gaming (17). These consistent findings indicate that altered gamma phasic synchrony is associated with hyperarousal in the sensory system as well as with an abnormal excitatory system. However, it remains unclear whether altered neural connectivity in patients with IGD is a trait marker or a state marker associated with the severity of IGD. A few studies using EEG coherence have shown abnormalities in brain connectivity in individuals with substance use disorder (SUD), which has a brain mechanism similar to that of IGD (7, 18, 19). For example, long-term abstinent as well as non-abstinent alcohol-dependent participants showed increased bilateral, intrahemispheric, and posterior EEG coherence (18). Similarly, abstinent heroin-dependent individuals exhibited increased left intrahemispheric gamma coherence compared to HCs (19). These findings suggest that enhanced neural connectivity is not normalized after a long period of abstinence or treatment and may reflect an endophenotype for SUD. Therefore, longitudinal studies with patients with IGD could help us understand the pathophysiology of and develop treatment interventions for IGD.

To the best of our knowledge, no studies have investigated longitudinal changes in resting-state EEG coherence following treatment of patients with IGD. Thus, we investigated cortical connectivity associated with treatment responses in patients with IGD to understand its underlying mechanism and to elucidate whether the altered phasic synchrony in individuals with IGD is a state or a trait marker. Based on previous findings (16, 17, 20), we hypothesized that patients with IGD would exhibit increased fast-frequency coherence at baseline and that this neurophysiological index would be sustained even though their IGD symptoms improved after 6 months of outpatient management.

## Materials and methods

### Participants

This longitudinal study included 62 male participants aged 18–38 years who were recruited from the SMG-SNU Boramae Medical Center and the surrounding community in Seoul, Republic of Korea. Thirty patients were classified as having IGD based on the criteria of the Diagnostic and Statistical Manual of Mental Disorders, Fifth Edition and diagnosed by a clinically experienced psychiatrist (1). Thirty-two participants served as HCs. The present study included only those patients who spent more than 4 h/day and/or 30 h/week playing Internet games. Additionally, Young's Internet Addiction Test (Y-IAT) was used to assess the severity of IGD symptoms (21). Baseline clinical assessments and an EEG scan were performed on all participants. Since baseline assessments, 18 of the 30 patients with IGD who had comorbid depressive or anxiety symptoms continued pharmacotherapy with serotonin reuptake inhibitors (SSRIs) using the average daily doses: escitalopram at 15.83 ± 9.17 mg, fluoxetine at 50.00 ± 9.17 mg, or paroxetine at 30.00 ± 14.14 mg. No drugs other than SSRIs were used in this study. After 6 months of continued treatment, they completed follow-up assessments including clinical measures and EEG recording. The primary treatment outcome was the change in the IAT score from pre- to post-treatment. HC participants who played Internet games < 2 h/day were recruited directly from local communities. None of the participants had a history of intellectual disability, psychotic disorder, or neurological disorder, and all were right-handed. Participants with an estimated IQ of < 80 were excluded.

This study was approved by the Institutional Review Board of SMG-SNU Boramae Medical Center, Republic of Korea. All participants provided written informed consent after having received information about the study.

### EEG recordings

#### EEG data collection

Detailed information about the EEG recordings and data collection procedure were presented in our previous study (16). Resting-state EEG was recorded for 10 min (4 min with eyes closed, 2 min with eyes open, and 4 min with eyes closed) in an electrically shielded and soundproofed room with dim lights. Participants were instructed to relax and avoid any body movements and drowsiness. EEG activity was recorded from 64 electrodes based on the modified International 10–20 system in conjunction with vertical and horizontal electrooculograms and a mastoid reference electrode. The ground channel was located between the FPz and Fz electrodes. The EEG signals were recorded continuously using a 0.1–60 Hz online bandpass filter and a 0.1–50 Hz offline bandpass filter at a sampling rate of 1,000 Hz. Electrode impedances were kept at < 5 KΩ.

All EEG data were analyzed with NeuroGuide software (NG Deluxe 2.6.1, Applied Neuroscience; St. Petersburg, FL, USA) for the coherence analysis, and 19 of the 64 channels were driven by the NeuroGuide montage set as follows: FP1, FP2, F7, F3, Fz, F4, F8, T3, C3, Cz, C4, T4, T5, P3, Pz, P4, T6, O1, and O2. Artifacts due to eye blinks and movements during EEG recordings were eliminated by the automatic NG Deluxe 2.6.1 system and were visually detected.

#### Coherence

The coherence analysis methods were presented in Park et al. (16). To summarize, resting-state EEG data were transformed into the frequency domain using the fast Fourier transformation algorithm with the following parameters: epoch = 2 s, sampling rate = 128 samples/s (256 digital time points), frequency range = 0.5–40 Hz, and a resolution of 0.5 Hz with a cosine taper window to minimize leakage. The NG 2.6.1 program was used to obtain the coherence values. The accepted epochs of the EEG data were computed for each of the following frequency bands: delta (1–4 Hz), theta (4–8 Hz), alpha (8–12 Hz), beta (12–25 Hz), and gamma (30–40 Hz). Furthermore, the intrahemispheric coherence for each band was examined using the F3–C3, F3–T3, F3–P3, C3–T3, C3–P3, and T3–P3 electrode pairs on the left hemisphere and the F4–C4, F4–T4, F4–P4, C4–T4, C4–P4, and T4–P4 electrode pairs on the right hemisphere. Interhemispheric coherence was computed between electrode pairs F3–F4, C3–C4, T3–T4, and P3–P4.

### Psychological assessments

#### Wechsler adult intelligence scale

The Korean version of the Wechsler Adult Intelligence Scale was administered to all participants to calculate their IQ (22–24).

#### Questionnaires

The Korean version of all questionnaires have been validated (25–28).

##### Young's IAT (Y-IAT)

The Y-IAT was used to measure the severity of Internet addiction. All 20 items are rated on a five-point scale from 1 to 5. Thus, total scores range from 20 to 100 (21, 28). The Cronbach's alpha for this study was 0.97.

##### Beck depression inventory-II (BDI-II)

The BDI-II was administered to assess the severity of depressive symptoms (26, 29). Each item is rated on a four-point scale from 0 to 3, and total scores for all 21 items can range from 0 to 63. The Cronbach's alpha for this study was 0.95.

##### Beck anxiety inventory (BAI)

The BAI includes a total of 21 items and addresses the intensity of anxiety symptoms (25, 30). Responses are rated on a four-point scale, and scores range from 0 to 3. The total BAI score, which ranges from 0 to 63, is obtained by summing all 21 items. The Cronbach's alpha for this study was 0.94.

##### Barratt impulsiveness scale-11 (BIS-11)

The BIS-11, which was used to measure impulsivity (27, 31), is a 30-item self-report questionnaire that includes three subscales that measure impulsivity (attention, motor, and non-planning). Each item is rated on a four-point scale from 1 to 4. The Cronbach's alpha for this study was 0.79.

### Statistical analysis

The baseline demographic and psychological variables were analyzed by independent *t*-tests, whereas differences in the psychological variables before and after treatment were analyzed by paired *t*-tests. Separate generalized estimating equations (GEEs) were used to assess the group effects in the EEG data for each frequency band to examine the correlations among repeated measurements (32, 33). Intra- and interhemispheric coherence values were analyzed by the GEEs using the following factors at baseline and at the end of the 6-month outpatient treatment period, respectively: intrahemispheric coherence was analyzed according to group (IGD and HC) × region (fronto-central, fronto-temporal, fronto-parietal, centro-temporal, centro-parietal, and temporo-parietal) × hemisphere (left and right); and interhemispheric coherence was evaluated according to group (IGD and HC) × region (frontal, central, temporal, and parietal). In these analyses, we controlled for education and BDI-II, BAI, and BIS-11 scores to identify group differences. All statistical analyses were performed using SPSS 20.0 software (SPSS Inc., Chicago, IL, USA).

## Results

### Demographic and psychological variables before and after treatment

The patients with IGD did not differ from the HCs in terms of age or IQ. However, significant differences in education, BDI-II, BAI, and BIS-11 scores were observed between the two groups. The demographic and psychological characteristics of the IGD and HC groups are presented in Table 1. After 6 months of treatment, patients with IGD had significantly lower Y-IAT scores but not lower BDI-II, BAI, or BIS-11 scores compared with their baseline data (Table 2).

**Table 1:** Demographic and psychological characteristics of the study groups at baseline.

**Characteristics**	**IGD patients (*N* = 30)**	**Healthy controls (*N* = 32)**	***t***	***p***
Age (years)	23.27 ± 5.15	24.97 ± 3.70	1.50	0.139
Education (years)	12.93 ± 1.83	14.61 ± 2.39	3.044	0.004**
IQ	113.83 ± 13.22	119.53 ± 9.81	1.918	0.061
Y-IAT	69.27 ± 14.78	29.29 ± 8.53	−12.992	< 0.001***
BDI-II	18.45 ± 10.27	3.71 ± 3.89	−7.256	< 0.001***
BAI	15.24 ± 13.14	5.81 ± 5.43	−3.590	0.001**
BIS-11	68.69 ± 13.15	55.23 ± 8.87	−3.590	< 0.001***

Mean ± SD.

** p < 0.01.

*** p < 0.001.

IQ, Intelligence quotient; Y-IAT, Young's Internet Addiction Test; BDI-II, Beck Depression Inventory-II; BAI, Beck Anxiety Inventory; BIS-11, Barratt Impulsivity Scale-11.

**Table 2:** Changes in the clinical characteristics of patients with Internet gaming disorder (IGD) before and after treatment.

**Characteristics**	**Before (*N* = 18)**	**After (*N* = 18)**	***t***	***p***
Y-IAT	71.33 ± 12.12	58.80 ± 22.10	2.454	0.028*
BDI-II	19.31 ± 10.27	19.94 ± 14.67	−0.340	0.738
BAI	18.56 ± 15.51	18.19 ± 16.90	0.248	0.807
BIS-11	69.50 ± 10.16	67.86 ± 9.73	1.827	0.091

Mean ± SD scores for Y-IAT, BDI-II, BAI, and BIS-11.

* p < 0.05.

### EEG coherence

#### Baseline EEG coherence data

The statistical analysis using the GEEs of intrahemispheric coherence revealed significant main group effects in the beta and gamma bands at baseline after adjusting for demographic and psychological variables (Table 3). Specifically, patients with IGD [M (standard error of the mean; S.E.M.) = 48.95 (69.463)] exhibited significantly increased beta intrahemispheric coherence than did HCs [M (S.E.M.) = 41.68 (70.187)]. Patients with IGD [M (S.E.M.) = 58.65 (111.862)] also showed significantly higher coherence in the gamma band than did HCs [M (S.E.M.) = 46.03 (113.029)]. Additionally, an interaction effect was revealed for group × hemisphere. The IGD group [M (S.E.M.) = 49.11 (68.393)] had significantly increased delta intrahemispheric coherence in the right hemisphere compared to the HC group [M (S.E.M.) = 42.36 (69.106)]. An analysis of interhemispheric coherence did not reflect a significant main effect of group, an interaction effect of group × region, or a group × hemisphere interaction.

**Table 3:** Effects on EEG intrahemispheric coherence controlling for the effects of demographic (education) and psychological (scores on the BDI-II, BAI, and BIS-11) characteristics before and after treatment.

	**Comparison between IGD at pre-treatment and HC**			**Comparison between IGD at post-treatment and HC**		
	***χ^2^***	***p***	***Post hoc***	***χ^2^***	***p***	***Post hoc***
**DELTA**						
Group	1.958	0.162		0.436	0.509	
Group × Region	5.886	0.317		7.705	0.173	
Group × Hemisphere	6.625	0.010*	Right, IGD: pre > HC	1.343	0.246	
Group × Region × Hemisphere	6.661	0.757		5.453	0.859	
**THETA**						
Group	0.409	0.522		2.165	0.141	
Group × Region	4.089	0.537		8.568	0.128	
Group × Hemisphere	3.656	0.056		1.210	0.271	
Group × Region × Hemisphere	4.740	0.908		7.095	0.716	
**ALPHA**						
Group	1.074	0.300		0.527	0.468	
Group × Region	9.478	0.091		13.950	0.016*	
Group × Hemisphere	0.627	0.428		0.419	0.518	
Group × Region × Hemisphere	5.473	0.857		3.277	0.974	
**BETA**						
Group	7.058	0.008**	IGD: pre > HC	14.074	0.000***	IGD: post > HC
Group × Region	8.326	0.139		9.068	0.106	
Group × Hemisphere	0.186	0.667		0.002	0.964	
Group × Region × Hemisphere	6.104	0.806		6.563	0.766	
**GAMMA**						
Group	8.118	0.004**	IGD: pre > HC	6.355	0.012*	IGD: post > HC
Group × Region	0.972	0.965		2.709	0.745	
Group × Hemisphere	0.122	0.726		0.076	0.782	
Group × Region × Hemisphere	3.736	0.958		5.028	0.889	

* p < 0.05.

** p < 0.01.

*** p < 0.001.

#### Changes in EEG coherence data following treatment

**Figure 1:**
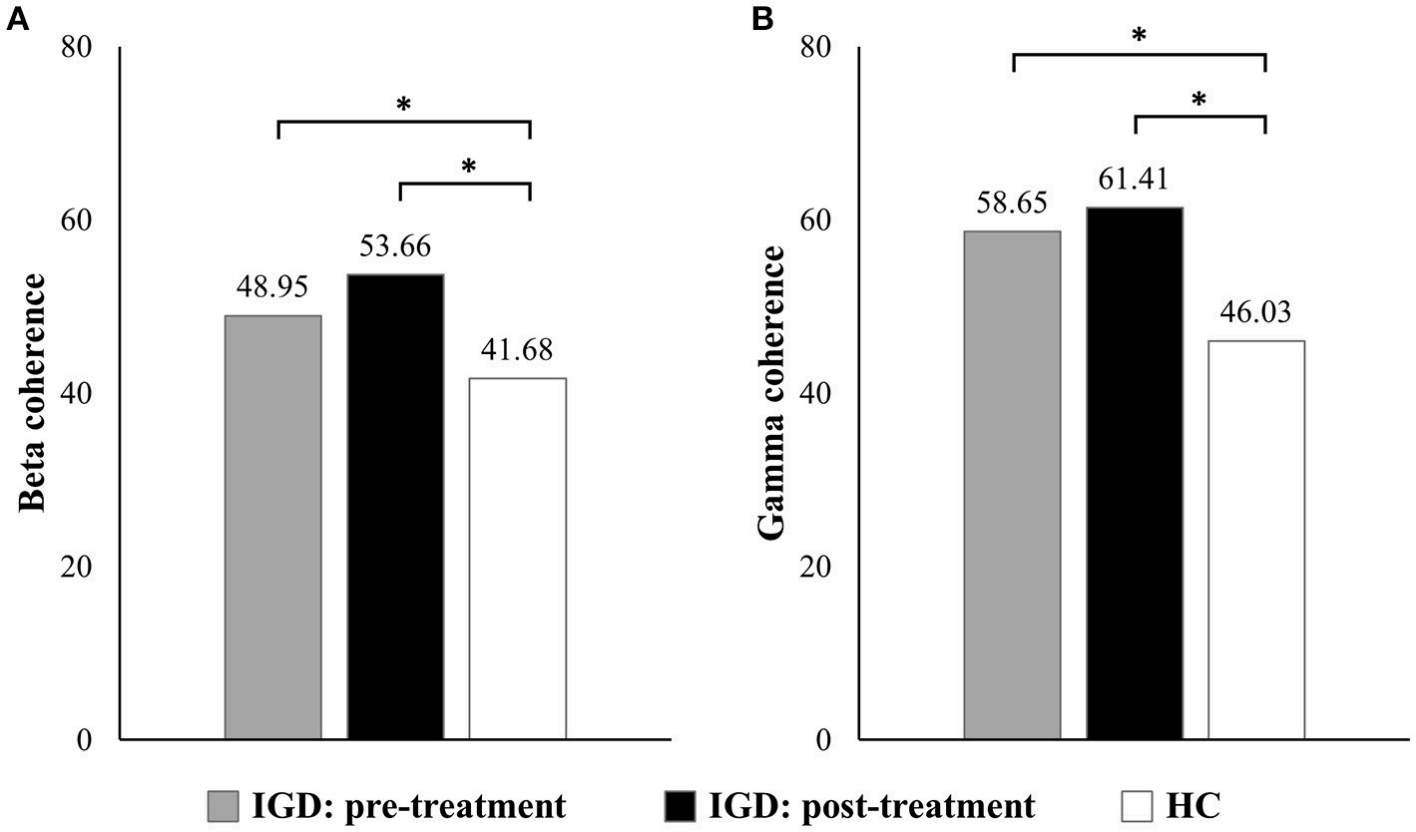
Main effects on EEG intrahemispheric **(A)** beta and **(B)** gamma coherence before and after treatment. *P < 0.05.

No significant EEG coherence changes were observed in any of the pre-treatment or post-treatment bands in the IGD group. However, a main effect of group was observed in beta and gamma coherence at the post-treatment assessment (Table 3 and Figure 1). Specifically, patients with IGD [M (S.E.M.) = 53.66 (75.338)] showed increased beta intrahemispheric coherence compared with HCs [M (S.E.M.) = 40.54 (77.143)]. Intrahemispheric coherence for the gamma band was significantly higher in patients with IGD [M (S.E.M.) = 61.41 (126.700)] than HCs [M (S.E.M.) = 46.51 (129.734)] at the post-treatment evaluation. Additionally, according to the post hoc analysis, there was an interaction effect of group × region in alpha coherence but no significant group differences.

## Discussion

To our knowledge, this is the first study to investigate longitudinal changes in neural connectivity measured by EEG coherence in patients with IGD. Participants with IGD exhibited increased intrahemispheric EEG coherence in the beta and gamma bands at baseline. However, these abnormal phase synchrony patterns were not normalized after 6 months of pharmacotherapy, even though the patients with IGD showed significant improvements in their IGD symptoms. Accordingly, our results indicate that increased beta and gamma coherence during the resting state may be an important neurophysiological trait marker of patients with IGD.

The IGD group showed significantly greater fast-frequency intrahemispheric coherence than did the HC group at baseline. Beta band activity on the resting EEG is considered to predispose a patient to substance use and is an electrophysiological marker of hyperexcitability due to an excitation–inhibition imbalance in the brain (34, 35). Increased intrahemispheric beta coherence has been related to the vulnerability factor for IGD (17, 36). For example, Youh et al. (17) showed that increased beta coherence in the frontotemporal area was more common in patients with comorbid IGD and major depressive disorder (MDD) compared to patients with only MDD. The authors suggested that enhanced beta coherence may reflect excessive online gaming and indicate the altered neural synchronization between brain regions in patients with IGD.

The increased EEG gamma coherence before treatment is consistent with a previous study (16). Gamma activity is commonly thought to reflect a variety of neural functions, including response inhibition and distribution of attentional resources (37–40). Our research group has reported that increased gamma intrahemispheric coherence is related to dysfunctional impulse control, the reward system, and the severity of IGD symptoms (16). Furthermore, Choi et al. (41) determined that increased gamma activity during a resting state is related to inhibitory impairment and trait impulsivity in patients with IGD. Taken together, these findings suggest inefficient neural synchrony and functional connectivity in patients with IGD.

After 6 months of outpatient management, the patients with IGD exhibited improvements in their IGD symptoms compared with baseline, but they still showed increased beta and gamma intrahemispheric coherence compared with HCs. A few studies conducted using SSRIs reported that pharmacotherapy reduces IGD symptoms (20, 42). Serotonin is thought to play an important role in depression, anxiety, and impulsivity (43). Therefore, treatment with an SSRI appears to be effective in reducing the severity of IGD. However, the present study did not find improvements in altered intrahemispheric coherence in the beta and gamma bands after 6 months of SSRI treatment. These findings suggest that increased fast-frequency coherence can be considered a potential trait marker of IGD rather than a state marker.

The present study was subject to certain limitations. First, our results may be of limited generalizability because the number of participants in this study was relatively small and only male participants were included. Second, the present study utilized typical outpatient care rather than well-organized treatment modalities. However, this study focused on the changes in phase synchrony patterns in patients with IGD rather than the treatment effects. Thus, additional studies will be needed to elucidate the effect of specific pharmacotherapy treatment on the neurophysiological markers of patients with IGD. Third, all patients with IGD included in this study had comorbid symptoms of depression or anxiety, which may have had confounding effects. Thus, psychological covariates were controlled in the final analysis to control for these comorbid symptoms.

Overall, the present study found that, at baseline, patients with IGD had increased intrahemispheric coherence in the fast-frequency band compared to the HC group. However, this abnormal neural connectivity was sustained after 6 months of outpatient treatment, indicating that the increased beta and gamma coherence during the resting state can be a considered neurobiological marker for the pathophysiology of IGD. The present research will contribute to a better understanding of the neurophysiological networks underlying IGD.

## Author contributions

J-SC and SK conducted the design and concept of the study. SP conducted the analyses and led the writing of the manuscript. J-SC guided and supervised the writing of the manuscript. HR, J-YL, AC, and D-JK contributed to conducting the study.

### Conflict of interest statement

The authors declare that the research was conducted in the absence of any commercial or financial relationships that could be construed as a potential conflict of interest.
